# Computational Analysis of the Inhibition Mechanism of NOTUM by the ONIOM Method

**DOI:** 10.1021/acsomega.2c01044

**Published:** 2022-04-07

**Authors:** Ibrahim Yildiz, Banu Sizirici Yildiz

**Affiliations:** † Chemistry Department, Khalifa University, PO Box 127788, Abu Dhabi 00000, UAE; ‡ CIVE Department, Khalifa University, PO Box 127788, Abu Dhabi 00000, UAE

## Abstract

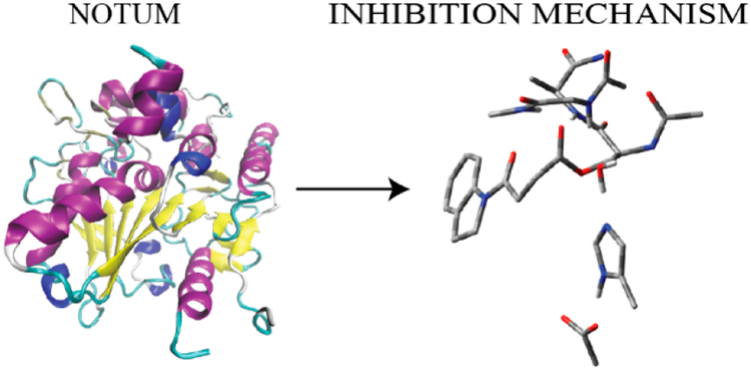

Notum is a member of serine hydrolyses that cleaves the palmitoleate moiety from Wingless-related integration site (Wnt) ligands. This enzyme plays crucial functions through modulating the Wnt signaling pathway. Inhibition of Notum carries therapeutic effects against a number of maladies including osteoporosis, cancer, and Alzheimer’s disease. Recently, a class of irreversible inhibitors based on esters of 4-(indolin-1-yl)-4-oxobutanoic acid have been reported. Using the crystal structures of enzyme-4-(indolin-1-yl)-4-oxobutanoate adduct and 4-(indolin-1-yl)-4-oxobutanoic acid-enzyme complex, we studied computationally the proposed inhibition mechanism using model systems based on the own n-layered integrated molecular orbital and molecular mechanics (ONIOM) method. In the first place, model systems were formulated to investigate the transesterification between the catalytic serine residue, Ser-232, and the methyl ester of 4-(indolin-1-yl)-4-oxobutanoate. In the second place, the hydrolysis mechanism of the resultant enzyme–inhibitor adduct was studied. The energetics of these steps were analyzed using a density functional theory functional in the ONIOM method. In addition, the roles of active-site residues during these steps were highlighted. It was found that the hydrolysis of the covalent adduct is highly endergonic corroborating the irreversible inhibition mechanism. These results will shed light not only on the inhibition mechanism but also on the catalytic mechanism.

## 1 Introduction

Serine hydrolyses consisting of numerous enzymes catalyze the hydrolysis of various substrates including proteins, peptides, and small molecules.^1^ It is divided into two main subgroups; serine proteases such as subtilisin, trypsin, and chymotrypsin act as digestive enzymes; metabolic serine hydrolyses catalyze the breakdown of ester and related functional groups.^2^ Recently, Notum has been shown to belong to the metabolic serine hydrolyze group, activating Wingless-related integration site (Wnt) proteins by hydrolyzing O-linked palmitoleate.^3,4^ By modulating the Wnt signaling, Notum plays important roles in many processes such as fat metabolism,^5^ bone strength,^6^ neurogenesis,^7^ etc. In addition, Wnt signaling was found to be dysregulated in Alzheimer’s disease.^8^ A recent study showed that increased Wnt signaling is associated with colorectal cancer.^9^ On the basis of these results, Notum appears as an important therapeutical target for a number of diseases.

**Figure 1:**
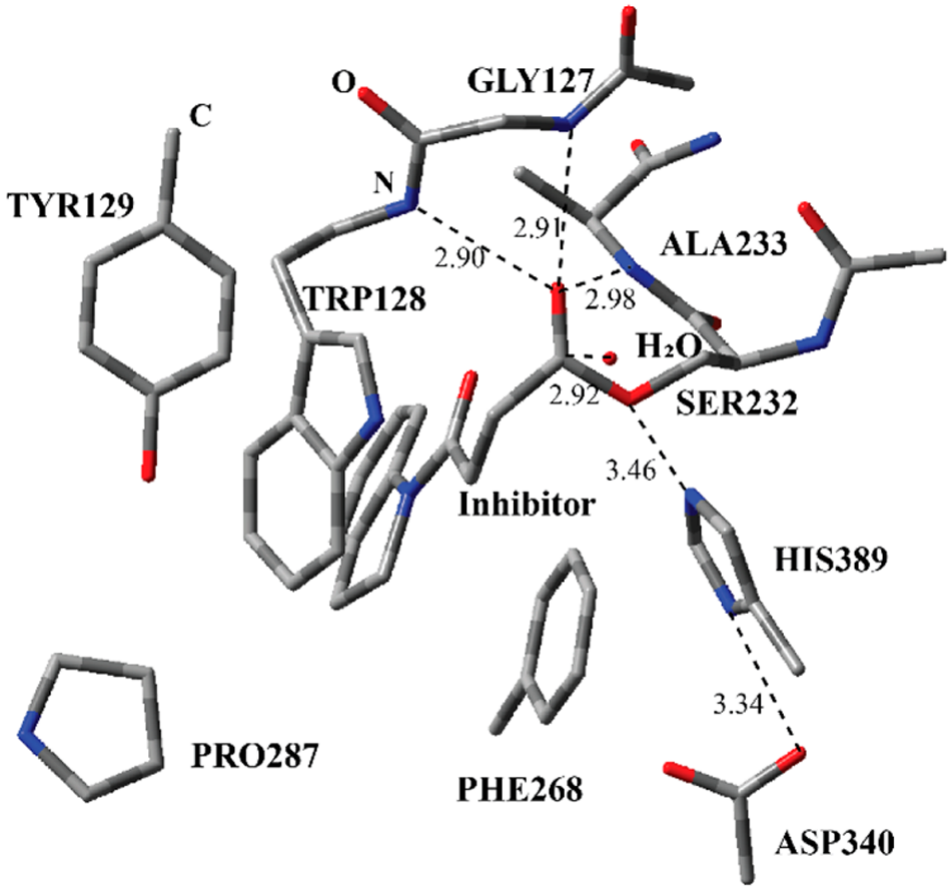
Structure of the active site of Notum covalently bound with the indole-based inhibitor through Ser232 together with important residues. The distances are given in angstroms.

Some studies reported reversible Notum inhibitors such as heteroaryl-fused thiophenes,^10,11^*N*-hydroxyhydantoin carbamates,^12^ and 1,2,3-triazole-based molecules.^13^ Recently, Zhao et al. has reported an irreversible Notum inhibitor, methyl 4-(indolin-1-yl)-4-oxobutanoate.^14^ The crystal structure of the inhibitor-enzyme complex showed a covalent adduct formation between catalytic Ser232 residue and inhibitor through an ester bond (Figure 1). The indole part of the inhibitor is surrounded with Trp128 and Phe268 through π-stacking interactions. These two residues together with Pro54 and Tyr129 form a hydrophobic pocket around the indole fragment of the inhibitor. The ester carbonyl O of the inhibitor is flanked with the amide N of Trp128, Gly127, and Ala233 suggesting H-bonding interactions. The covalent adduct shows the Ser-His-Asp catalytic triad common for the Serine protease family.^2^ The H-bonding network among Ser232-His389-Asp340 suggests the common reaction mechanism for the carboxylesterase family.^15^

**Figure 2:**
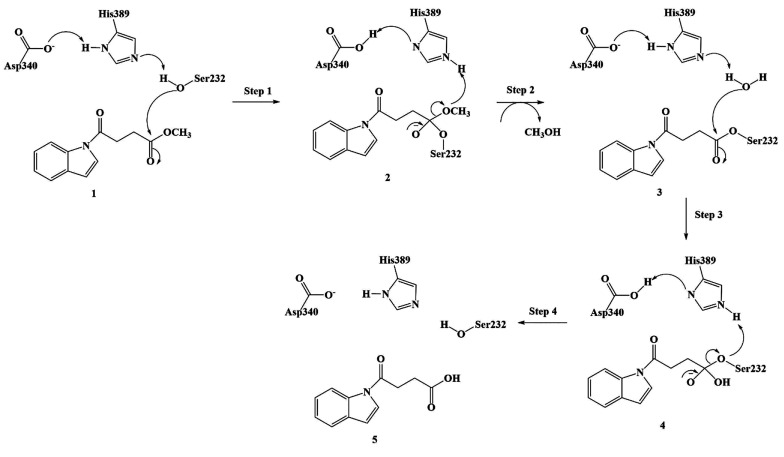
Proposed inhibition mechanism of Notum (Step 1 and Step 2) by indole-based ligand **1**.

On the basis of this, the inhibition of Notum was proposed to result from the formation of the irreversible covalent adduct between Ser232 and inhibitor **1** as a result of the ester bond formation^14^ (Step 1 and Step 2 in Figure 2). On the basis of the crystal structure of the adduct (Figure 1), it was stated that the deacylation process (Step 3 and Step 4 in Figure 2) corresponding to the hydrolysis of adduct **3** into the carboxylic acid (**5** in Figure 2) and free Ser232 is hindered due to an unfavorable positioning of the water molecule together with the strong hydrophobic interaction of the inhibitor with the surrounding residues.

Various computational studies elucidating the reaction mechanism of the esterase family have been reported.^16^ Zhang et al. studied the acylation process catalyzed by acetylcholinesterase (AChE) with a hybrid quantum mechanics-molecular mechanics (QM-MM) method.^17^ It was found that, as the His447 abstracts a proton from Ser203, Ser203 forms a tetrahedral oxyanion stabilized by H-bonding interactions. Liu et al. reported the reaction mechanism of cocaine esterase (CocE), which catalyzes the hydrolysis of (−)-cocaine.^18^ Using QM-MM calculations, it was established that first the catalytic Ser residue forms a tetrahedral intermediate with the ester substrate; second, this intermediate dissociates to form a new ester between the Ser residue and (−)-cocaine; third, a catalytic water molecule adds to the carbonyl carbon of the (−)-cocaine ester to form a tetrahedral oxyanion; finally, this intermediate breaks down into the carboxylic acid form of the (−)-cocaine and Ser residue. Aranda et al. studied the reaction mechanism of plant carboxylesterase AeCXE1 (Actinidia eriantha) with propyl acetate as substrate using density functional theory (DFT) calculations.^19^ It was reported that the acylation (Steps 1 and 2 in Figure 2) and deacylation (Steps 3 and 4 in Figure 2) processes proceeded in four steps involving tetrahedral oxyanion intermediates. Goegheri et al. used DFT calculations to study the hydrolysis of two esters, namely, acetylcholine and methylcaprylate, by Candida Antarctica lipase B (CALB).^20^ It was shown that the oxyanion intermediates are stabilized by the surrounding residues.

To the best of our knowledge, up to now both the catalytic and inhibition mechanisms of Notum have not been studied computationally. Furthermore, computational mechanistic studies of the inhibition mechanism of enzymes are rare, and they could potentially elaborate the catalysis mechanism. We manipulated QM-MM hybrid calculations to highlight electronic as well as noncovalent factors for the inhibition process of Notum. Insights into the mechanistic details of Notum could potentially help design new therapeutics for a variety of diseases. In this study, the proposed acylation and deacylation processes of the Notum inhibition mechanism were studied with the own n-layered integrated molecular orbital and molecular mechanics (ONIOM) method consisting of QM-MM calculations using crystal structures Notum complexed with **1** and **5** (Figure 2). The possible reaction pathways were constructed using relaxed potential energy surface (PES) scans employing the coordinates of bonds forming and breaking. On the basis of these scans, model enzyme-reactant complex, transition states, intermediate complexes, and enzyme–product complex were obtained. These models highlighted the roles of active-site residues during the inhibition process. In addition, the activation barriers in terms of Gibbs free energies were evaluated.

## 2 Computational Details and Methodology

In the calculations ONIOM^21^ method within Gaussian 09 package^22^ was used to study the Notum inhibition mechanism. In the Quantum Mechanics (QM) layer, the M06-2X^23^ DFT functional, while in the MM (Molecular Mechanics) layer, AMBER force field^24^ was utilized. The M06-2X functional has been shown to produce good results in the main-group chemistry.^25^ Amber 94 MM charges were used for all residues. The mechanical embedding option was included in the calculations. None of the coordinates were frozen in QM and MM regions. The restrained electrostatic potential (RESP) charges of atoms in **1**, **2**, **3**, **4**, and **5** (Figure 2) were calculated with the HF/6-31G(d) method, and their MM parameters were obtained with the antechamber option in AMBER 16.^26,27^ For the other standard amino acid residues, the built-in Amber 94 MM charges were used.

The optimized geometries of the reactant-complex (RC), intermediate complexes (IC), transition state (TS), and products-complex (PC) were obtained using the 6-31G basis set. Single-point energy calculations were performed using a larger basis set, 6-311+G(2d,2p). The TS structures were characterized with one negative eigenvalue, and RC and PC structures did not have any negative eigenvalues in frequency calculations. The TS structures were estimated through relaxed potential energy surface (PES) scans. In these scans, the bonds that are forming or breaking were scanned, and the maximum energy points were chosen for TS optimization using the Berny algorithm.^28^ RC, IC, and PC complexes were optimized choosing appropriate points in the PES scans.

**Figure 3:**
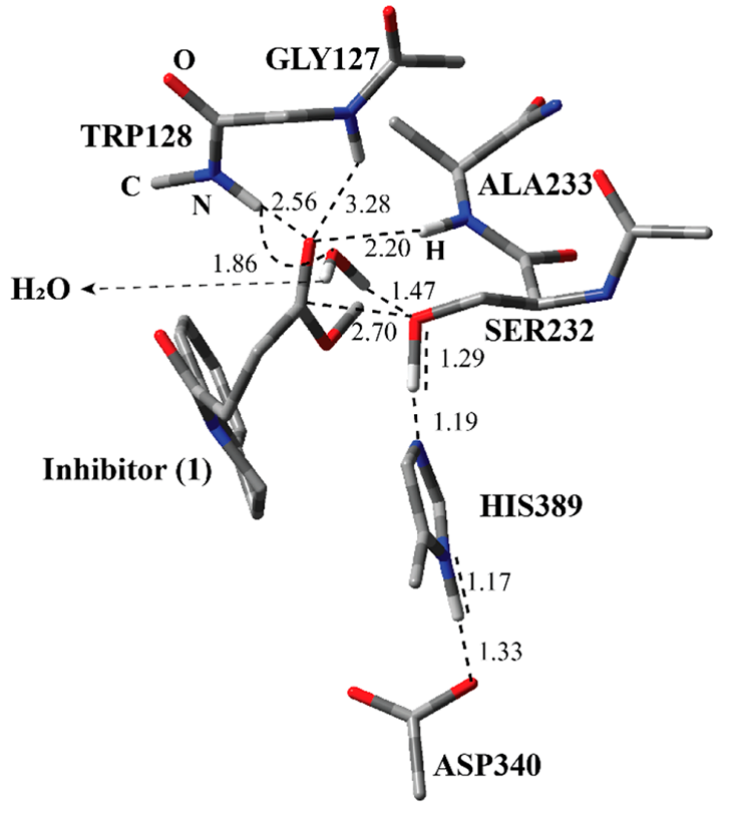
Structure of optimized RC including the inhibitor **1**, a water molecule, and catalytically important residues in the QM region belonging to the first step of the acylation process (Step 1 in Figure 2) obtained with ONIOM (M06-2*X*/6-31G: Amber) with tube models excluding H atoms except the ones shown with ivory color. The distances are given in angstroms.

**Figure 4:**
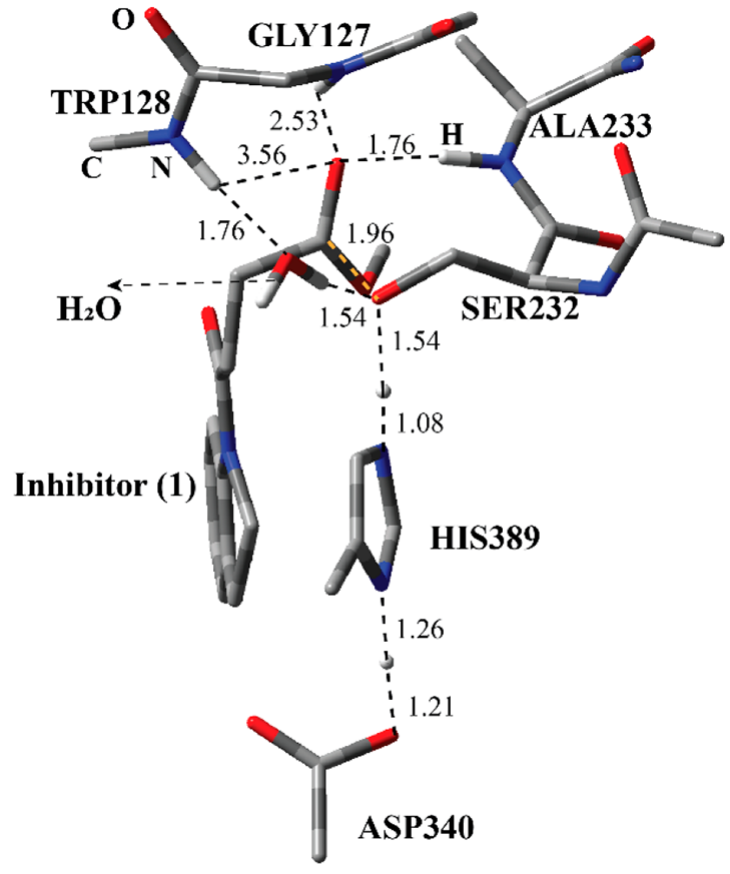
Structure of optimized TS-1 including inhibitor **1**, a water molecule, and catalytically important residues in the QM region belonging to the first step of the acylation process obtained with ONIOM (M06-2*X*/6-31G:Amber) with tube models excluding H atoms except the ones shown with an ivory color. The distances are given in angstroms.

For the calculations involving the acylation process (Steps 1 and 2 in Figure 2), a model enzyme–substrate complex was generated from the crystal structure of Notum with **5** (PDB accession code: 7B37)^14^ using the VMD program.^29^ This complex was found inactive and provided a starting point to obtain a reactive enzyme–substrate complex. To this end, the carboxylic acid **5** (Figure 1) was converted manually into the methyl ester **1**. The model systems included inhibitor **1** and residues around it in a radius of 10 Å. The model system consisted of 985 atoms and 80 residues including 7 water molecules. Acetyl and N-methyl groups were incorporated into the N-terminal and C-terminal residues on the peripheries to maintain the original electrostatic environment surrounding the active site. The protonation states of the residues with ionizable groups were determined using the PropKa program.^30^ The total charge of the ONIOM models was −4. In the ONIOM model systems, the QM region (Figure 4) included Ser232, His389, Asp340, Ala233, Gly127, Trp128 (amide part), a water molecule, and the amide groups of the residues of 126 and 234. The QM region included 95 atoms together with a total charge of −1. Part of the QM region residues were included into the MM region, and Figure 3 shows the QM region of these residues. For the calculations involving the deacylation process (Step 3 and Step 4 in Figure 2), the model enzyme–substrate covalent adduct was generated from the crystal structure of Notum covalently modified with inhibitor **1** (PDB accession code: 7ARG)^14^ following the same protocol as outlined for the acylation process.

The initial model structures obtained from the crystal structures for acylation and deacylation processes were optimized using the M06-2X functional with a 6-31G basis set. A PES scan was performed using the distance between the O atom of the Ser232 and the ester carbonyl of substrate **1** for the acylation process to locate the structures of RC, TS, and IC complexes (Steps 1 and 2 in Figure 2). In the same fashion, another PES scan was performed using the distance between the O atom of the catalytic water molecule and the ester carbonyl of the Ser232-inhibitor adduct (**3** in Figure 2) for the deacylation process to locate the IC, TS, and PC structures. Single-point energy calculations using optimized geometries obtained with the 6-31G basis set were performed with the 6-311+G(2d,2p) basis set.

## 3 Results and Discussions

### 3.1 Acylation Step

#### 3.1.1 Reactant Complex (RC)

The optimized geometry of the RC (Figure 3) between inhibitor **1** and the active site residues reveals important interactions. Ser232 and inhibitor **1** are kept together through a series of H-bonding interactions. Three amide H atoms have close distances with the ester carbonyl O atom of inhibitor **1**. The amide groups of Ala233 and Trp128 are closer than that of Gly127. These three residues are poised to stabilize the transition state during the nucleophilic addition of Ser232 to the ester carbonyl atom of the inhibitor. The active-site water molecule has H-bonding interactions with Ser232 and Trp128. These interactions collectively keep Ser232 at a close distance to inhibitor **1**. In addition, the inhibitor is surrounded with the indole part of Trp128, Phe268, Pro54, and Tyr129 similar to the crystal structure of the covalent adduct in Figure 2. (All of the coordinates are provided in the Supporting Information.) However, these residues were placed in the MM region and, for the sake of simplicity, are not shown in Figure 4. Another striking observation is that the distance between the H and O atoms of the hydroxyl group in Ser232 is considerably longer than a normal O–H bond, and the H atom is closer to the N-3 atom of His389. This suggests that His389 has already started to act as the catalytic base to deprotonate the hydroxyl group of Ser232 in RC. It clearly indicates the roles of the Ser-His-Asp catalytic triad in the acylation process. Furthermore, the H atom at the N-1 position of His389 has a close H-bonding interaction with the carboxylate O of the Asp340. The distance between N-1 and the H atom in His389 is longer than that of an expected N–H bond length, and the H atom is considerably close to Asp340. This observation implies that, in the catalytic triad, Asp340 acts as a general base to deprotonate the N-1 position of His389, which in turn becomes more basic and deprotonates the hydroxyl group of Ser232, which in turn becomes more nucleophilic.

#### 3.1.2 Transition State-1 (TS-1)

The optimized geometry of the transition state for the first step of the acylation process (Step 1 in Figure 2) (TS-1) reflects the function of the catalytic triad in a more pronounced way (Figure 4). Ser232 is almost deprotonated by His389. By the same token, the proton at the N-1 position of His389 is closer to Asp340. Ser232 is now in a close proximity to the ester carbonyl of the inhibitor. Through two H-bonding interactions, the water molecule together with Trp128 holds Ser232 close to the inhibitor as in the case of RC-1. The H atoms on the amide nitrogen of Ala233 and Gly127 residues are closer to the forming oxyanion on the inhibitor as compared to RC-1. This suggests that, by H-bonding interactions, they stabilize the forming oxyanion. Although the amide group of Trp128 moved further away from the oxyanion, it is closer to stabilize the oxyanion.

**Figure 5:**
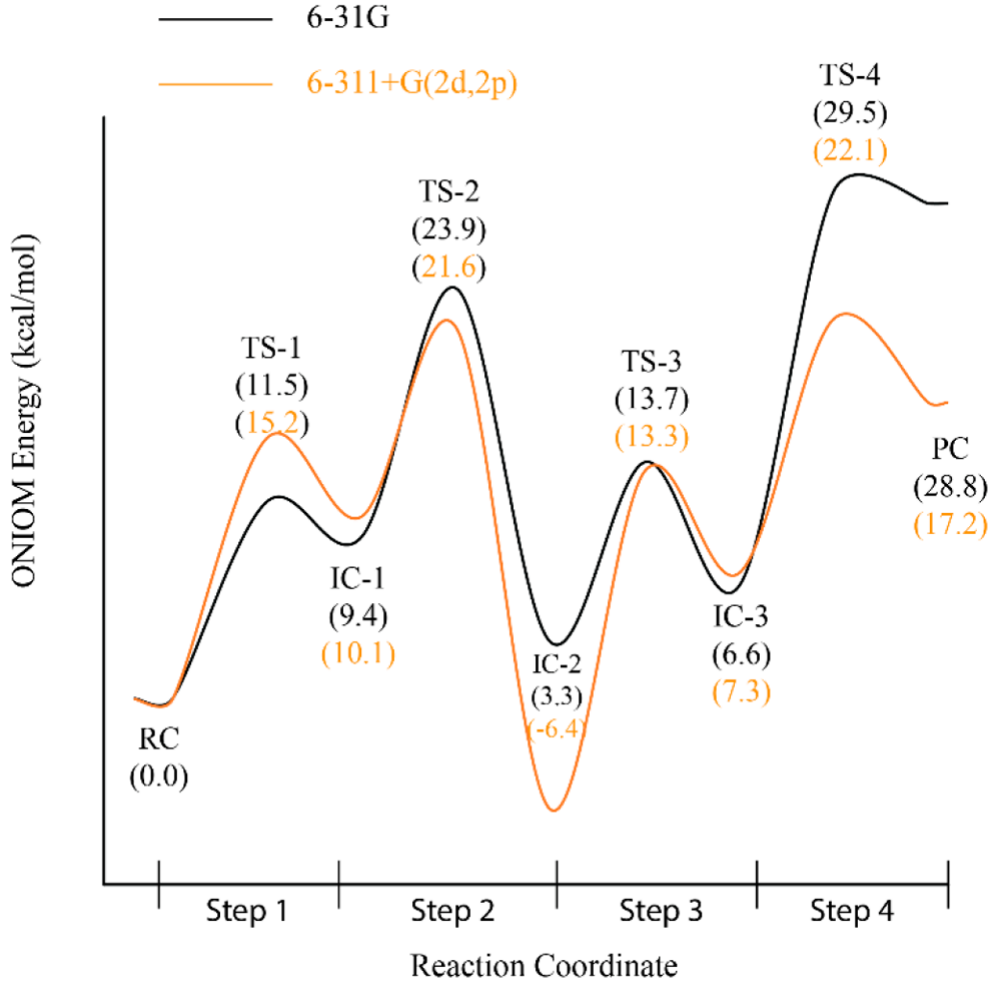
Energy profile for the acylation (Steps 1 and 2) and deacylation (Steps 3 and 4) processes of inhibitor **1** by Notum obtained with the ONIOM (M06-2*X*/6-31G:Amber) method (The values in the parentheses with black color refers to the ONIOM electronic energies of each complex (in kcal/mol) relative to the initial RC obtained with optimization with 6-31G basis, and the values in the parentheses with an orange color refer to the ONIOM electronic energies of each complex (in kcal/mol) relative to the initial RC obtained with a single-point energy calculation using the 6-311+G(2d,2p) basis set).

The ONIOM activation energy in terms of the electronic energy difference between TS-1 and RC was calculated; the value is 11.5 kcal/mol (Step 1 in Figure 5). A similar value (10.5 kcal/mol) was obtained based on free energies (Figure S1). Single-point energy calculations using a larger basis set, 6-311+g(2d,2p), using optimized geometries obtained with the 6-31G basis set revealed a slightly higher energy barrier (15.2 kcal/mol).

#### 3.1.3 Intermediate Complex-1 (IC-1)

**Figure 6:**
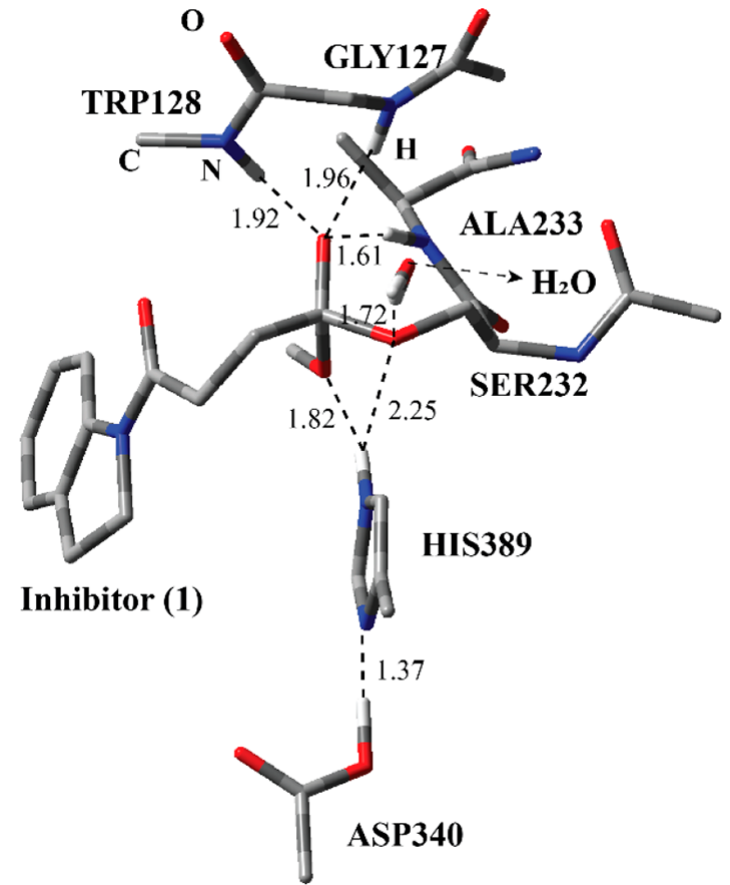
Structure of optimized IC-1 including the inhibitor **1-**Ser232 adduct, a water molecule, and catalytically important residues in the QM region belonging to the first step of the acylation process obtained with ONIOM (M06-2*X*/6-31G:Amber) with tube models excluding H atoms except the ones shown with an ivory color. The distances are given in angstroms.

The optimized structure of the intermediate complex (IC-1) for the nucleophilic addition of Ser232 to inhibitor **1** revealed a tetrahedral oxyanion species stabilized by three H-bonding interactions (Figure 6). The O atom in Ser232 is fully deprotonated and connected to the ester carbonyl C of inhibitor **1** forming a tetrahedral intermediate (**2** in Figure 2). The resulting oxyanion has close H-bonding interactions with Trp128, Ala233, and Gly127 residues through their amide groups. The water molecule in the active site moved away from Trp128 and has H-bonding interactions with the O atom at the Ser232 residue. The H atom at the N-3 position of His389 has H-bonding interactions both with the O atom at the methyl ester part of the inhibitor and the O atom at Ser232. Asp340 is in a protonated state, and this proton has an H-bonding interaction with the N-1 atom at His389.

The structure of the IC-1 clearly shows that the acylation process does not proceed through a single-step substitution. A second step is required for the loss of methanol, which will form a planar sp^2^ ester carbonyl group. The energy difference between TS-1 and IC-1, which is the activation energy of the reverse process, was calculated (2.06 kcal/mol) in terms of the ONIOM energy with a 6-31G basis set (Figure 5). The same barrier was estimated (4.57 kcal/mol) with the 6-311+g(2d,2p) basis set. These results indicate that the first step is an endergonic process and that the intermediate is a reactive species.

#### 3.1.4 Transition State-2 (TS-2)

**Figure 7:**
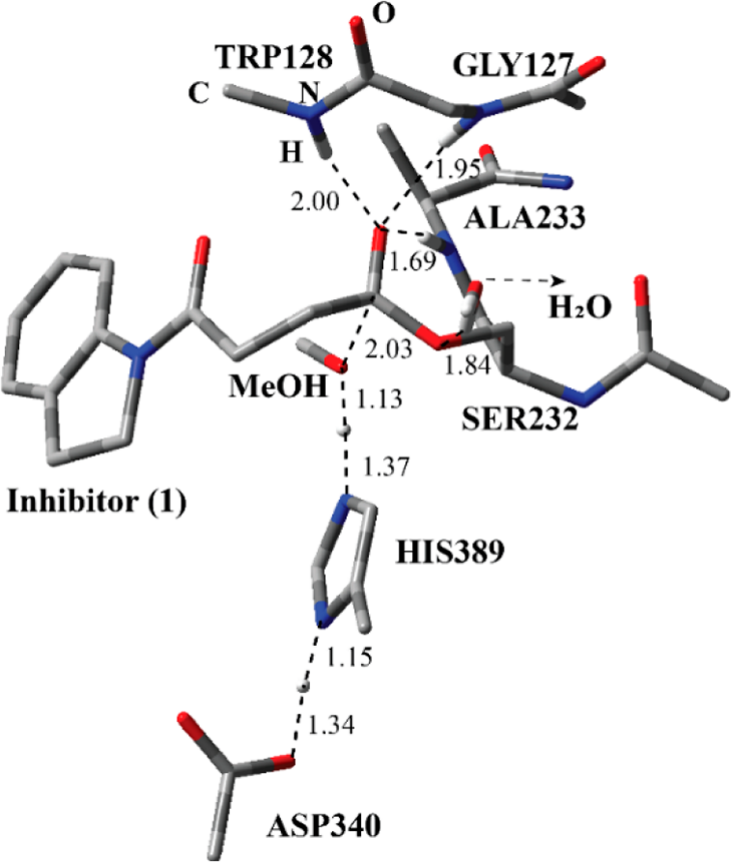
Structure of optimized TS-2 including the inhibitor **1-**Ser232 adduct, a water molecule, and catalytically important residues in the QM region belonging to the second step of the acylation process obtained with ONIOM (M06-2*X*/6-31G:Amber) with tube models excluding H atoms except the ones shown with an ivory color. The distances are given in angstroms.

The optimized structure of the IC-1 for the nucleophilic addition of Ser232 to the inhibitor **1** (Figure 6) revealed the tetrahedral oxyanion species stabilized by three H-bonding interactions. According to the proposed mechanism (Steps 1 and 2 in Figure 2), Ser232 substitutes methanol at the inhibitor. In order to model the leaving of methanol, a new PES scan was conducted by scanning the distance between the carbonyl C and the O atom of methyl ester part in the oxyanion (Figure 6). The highest energy point was subjected to TS optimization, and the resulting structure corresponded to the transition state (TS-2) for the second step of the acylation process (Figure 7). The optimized TS-2 structure reveals that, as the oxyanion intermediate loses its methoxy part, His389 protonates this leaving group (MeOH in Figure 7). At the same time, Asp340 starts to protonate the N-1 position at His389. Furthermore, the three residues Gly127, Trp128, and Ala233 still act as oxyanion hole residues by close H-bonding interactions through their H atoms at their amide N atoms. The water molecule has a H-bonding interaction with the O atom at Ser232 residue.

The energy difference between TS-2 and IC-1 corresponding to the ONIOM activation energy of the second step of acylation process was calculated (14.5 kcal/mol) with the 6-31G basis set (Step 2 in Figure 5). This value is estimated (9.6 kcal/mol) in terms of free energy (Step 2 in Figure S1). Free energy calculations yielded a lower energy barrier for the second step of the acylation process by estimating a higher energy for IC-2 and a lower energy for TS-2 as compared to electronic energy calculations. This can be also observed as intersections of electronic and free energy curves in Figure S1 for Step 2. This indicates the contribution of entropic effects to the activation energy. The same barrier is estimated (11.0 kcal/mol) with the single-point energy calculations using the 6-311+g(2d,2p) basis set.

#### 3.1.5 Intermediate Complex-2 (IC-2)

**Figure 8:**
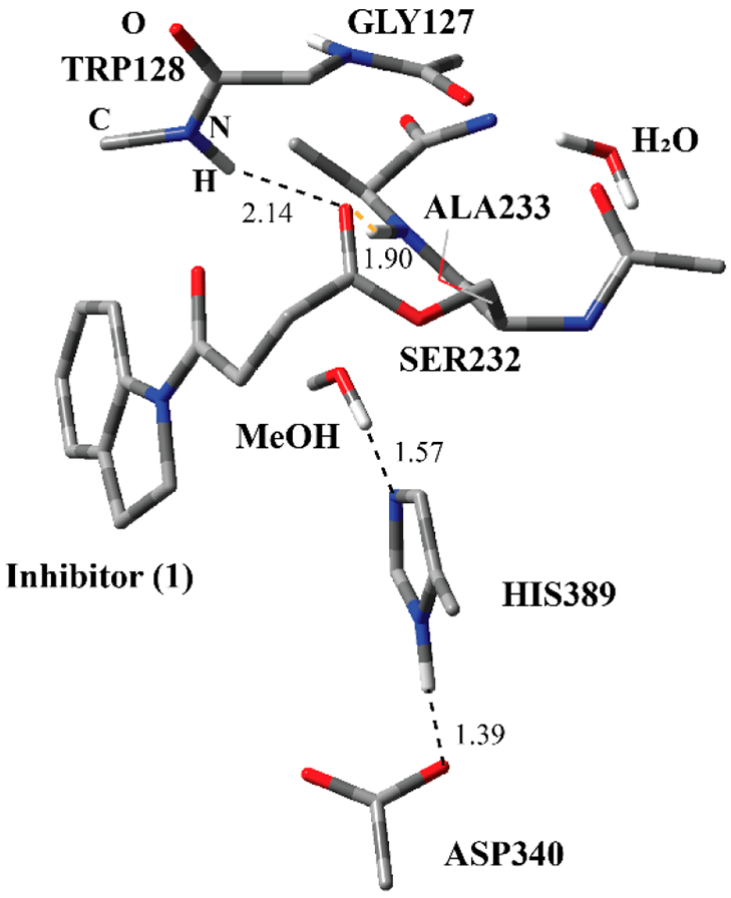
Structure of optimized IC-2 including the inhibitor **1-**Ser232 adduct, MeOH, two water molecules, and catalytically important residues in the QM region belonging to the second step of the acylation process obtained with ONIOM (M06-2*X*/6-31G:Amber) with tube models excluding H atoms except the ones shown with an ivory color. The second water molecule belonging to the MM region is shown in wireframe. The distances are given in angstroms.

A proper downhill point in the PES scan of the second step of the acylation process was chosen for the optimization of the second intermediate complex (IC-2) corresponding to the loss of a methanol molecule. The optimized structure of the IC-2 (Figure 8) shows that a methanol molecule was eliminated upon the protonation of the methyl ester by His389, and the ester formation between the inhibitor and Ser232 is complete. Trp128 and Ala233 still have preserved their H-bonding interactions with the ester carbonyl O atom of the inhibitor. However, the H-bonding interaction with the Gly127 is lost. His 389 is protonated by Asp340 at the N-1 position. As the methanol leaves, the water molecule having a previous H-bonding interaction with the O atom at Ser232 moved away, and another water molecule in the vicinity approached the carbonyl C atom of the ester. This water molecule, which is in the MM region, will act as the nucleophile responsible for the deacylation process, which will be discussed in the following section.

The ONIOM energy difference between TS-2 and IC-2 corresponding to the backward activation energy of the second step of the acylation process was calculated (20.6 kcal/mol) using the 6-31G basis set (Step 2 in Figure 6). The same barrier was estimated (30.6 kcal/mol) using single-point energy calculations with the 6-311+g(2d,2p) basis set (Step 2 in Figure 5). Calculations involving the 6-311+g(2d,2p) basis set predicted a more stable IC-2 structure, and the overall acylation process turned out to be an exergonic process. This result is in agreement with the experimental results^14^ in that the crystal structure of Notum together with inhibitor **1** resulted in the irreversible covalent adduct, which correspond to an IC-2 structure. Of all the calculated complexes, IC-2 is predicted to be the most stable species. Its low energy level predicted by the 6-311+g(2d,2p) basis set indicates the irreversible nature of the covalent adduct.

### 3.2 Deacetylation Process

On the basis of the proposed mechanism, following the acylation process, the deacylation process occurs as a result of the nucleophilic attack of a water molecule resulting the hydrolysis of the ester intermediate into a carboxylic acid form of the inhibitor and the free alcohol form of Ser232 (Steps 3 and 4 in Figure 2). The calculations involving the deacylation process utilized the crystal structure of the covalent adduct between Notum and inhibitor **1** (Figure 1). It was also possible to utilize the IC-2 (Figure 8) structure, which is the “calculated” model form of the inhibitor-enzyme covalent adduct excluding the MeOH molecule, which is still present in the active site. It is expected that the MeOH molecule will leave the active site following its release and that the catalytic triad will reorganize for the deacylation process. Considering these factors, for the calculations we did not continue with the calculated form (Figure 8). We opted to use the available crystal structure (Figure 1) belonging to the enzyme–inhibitor covalent adduct without a methanol molecule.

#### 3.2.1 Reactive Intermediate Complex-2 (r-IC-2)

**Figure 9:**
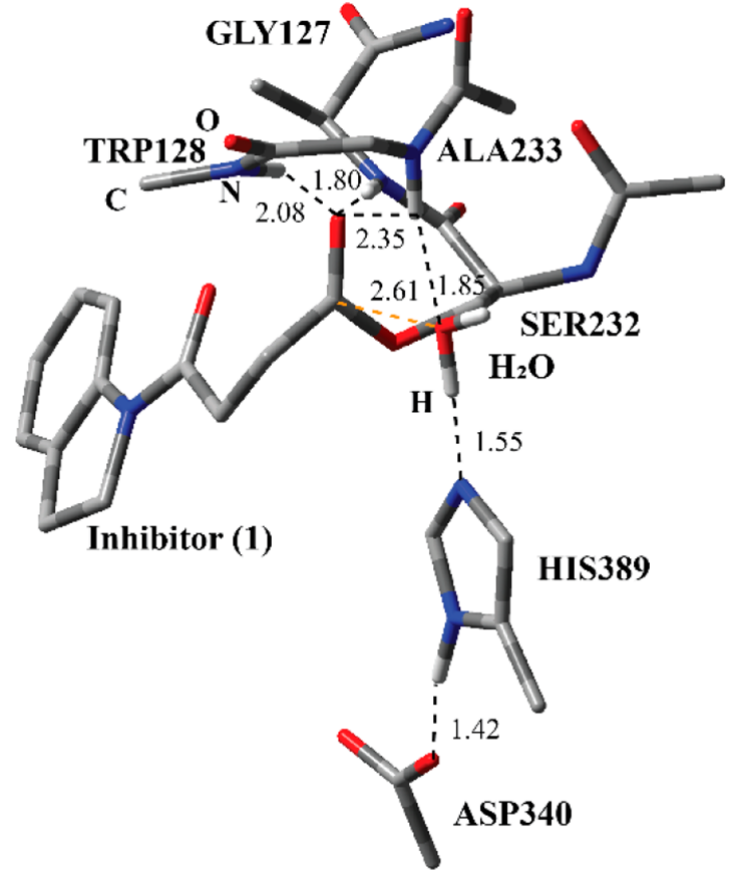
Structure of the optimized r-IC-2 for the first step of the deacylation process including the inhibitor **1-**Ser232 adduct, water molecule, and catalytically important residues in the QM region obtained with ONIOM (M06-2*X*/6-31G:Amber) with tube models excluding H atoms except the ones shown with an ivory color. The distances are given in angstroms.

Similar to the acylation process, PES scans were utilized to locate the TS, IC, and PC structures. The distance between the O atom in the water molecule and the carbonyl C atom of the ester was scanned to locate the TS structure for the hydrolysis of the ester. The optimized structure of the reactive intermediate complex (r-IC-2) (Figure 9) revealed a similar chemical environment as compared to the reactant complex (RC) (Figure 3) for the acylation step. Now in this step, instead of Ser232 acting as the nucleophile, the water molecule acts as the nucleophile. His389 is positioned to act as the general base to deprotonate the water molecule, while Asp340 is expected to act as the general base to deprotonate the N-1 position at His389. Through their amide H atoms, Trp128, Gly127, and Ala233 have a H-bonding interaction with the inhibitor’s ester carbonyl C, while Ala233 interacts with the nucleophilic water molecule. These three residues are expected to act as the oxyanion hole residues during and after the nucleophilic addition of the water molecule.

#### 3.2.2 Transition State-3 (TS-3)

**Figure 10:**
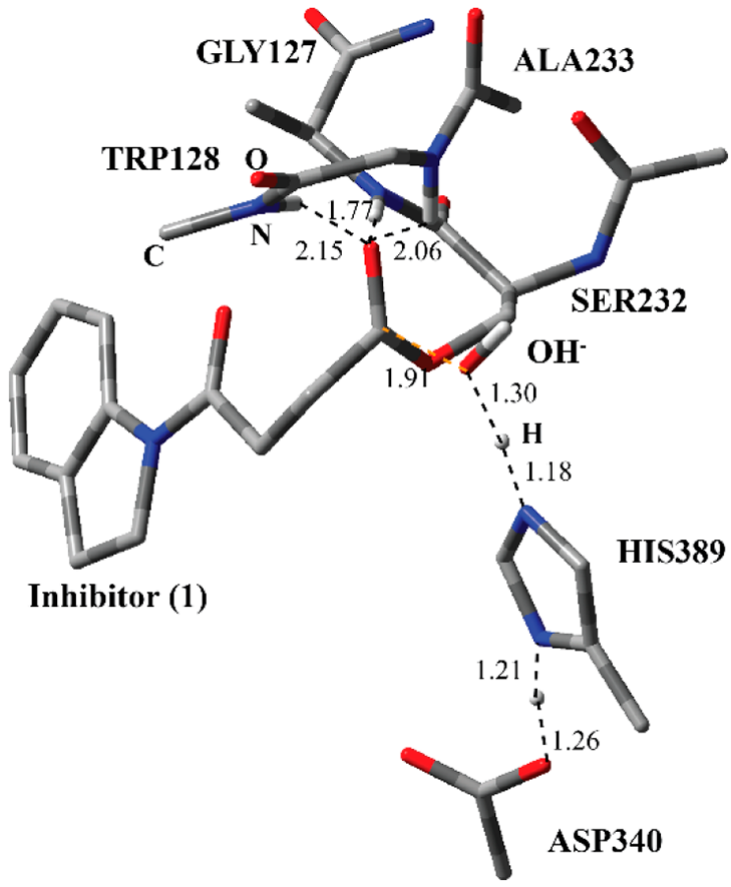
Structure of the optimized TS-3 structure for the first step of the deacylation process including the inhibitor **1-**Ser 232 adduct, water molecule, and catalytically important residues in the QM region obtained with ONIOM (M06-2*X*/6-31G:Amber) with tube models excluding H atoms except the ones shown with an ivory color. The distances are given in angstroms.

The optimized structure of the transition state leading to the nucleophilic addition of a water molecule (TS-3) (Figure 10) closely resembles, as expected, the transition state of the nucleophilic addition of the Ser232 to the inhibitor **1** molecule (Figure 4). As His389 deprotonates the water molecule forming an OH^–^ nucleophile, it approaches the carbonyl carbon. At the same time, a proton moves from His389 to Asp340. Three amide groups stabilize the forming oxyanion on the carbonyl O at the inhibitor.

The ONIOM energy difference between TS-3 and r-IC-2 corresponding to the activation energy of the first step of the deacylation process was calculated (10.5 kcal/mol) with the 6-31G basis set. Similarly, the same barrier is estimated (9.2 kcal/mol) in terms of free energy. Single-point energy calculations with the 6-311+G(2d,2p) basis set, however, estimated the value at 19.7 kcal/mol, which is almost 9 kcal/mol higher than that of the 6-31G basis set (Figure 5). In this step, the transition state corresponds to the formation of an oxyanion intermediate, which is expected to be a highly reactive species. In this regard, the estimated barrier with the 6-311+G(2d,2p) basis set seems more plausible. The energy levels of the TS-3 estimated both with 6-31G and 6-311+G(2d,2p) basis sets are similar (Figure 5). The reason why 6-311+G(2d,2p) estimated a higher barrier is that it estimated a more stable IC-2, which in turn resulted in a higher activation barrier. In addition, the energy levels of both TS1 and TS3 relative to RC (Figure 5) are estimated to be in similar ranges both by 6-31G and 6-311+G(2d,2p) basis sets. This can be attributed to the similar processes and interactions for both steps, which entail the formation of the oxyanion species. In both steps, a tetrahedral oxyanion intermediate forms with the same type of H-bonding interactions.

The addition of a H_2_O molecule to the carbonyl C of inhibitor **1** during the transition from r-IC-2 to TS-3 generates a similar tetrahedral complex as in the case of the transition from IC-2 to TS-2. That is to say, TS-2 and TS-3 represent similar transition-state structures with similar H-bonding interactions. In the case of TS-2, a methanol molecule is lost from the tetrahedral oxyanion complex, whereas, in the case of TS-3 a H_2_O molecule is added to form another tetrahedral oxyanion complex. The activation energies from IC-2 to TS-2 and from r-IC-2 to TS-3 are expected to have closer values. In other words, TS-2 and TS-3 should have similar energy levels as compared to IC-2. However, our calculations indicated that TS-2 has almost 10.2 kcal/mol more energy than TS-3 in terms of ONIOM energies with the 6-31G basis set (Figure 5). This much difference cannot be explained based on steric factors stemming from the relative sizes of H_2_O or methanol molecules. A plausible explanation can be deduced from the TS-2 (Figure 7) and TS-3 structures (Figure 10). Loss of methanol and the addition of a water molecule occur at the same carbonyl carbon from the opposite sides. Although the processes are similar, the chemical environments on both sides are different. This may cause considerable stability differences between TS-2 and TS-3. To this end, a control calculation was performed. The methyl group in TS-2 was converted manually to a H atom to obtain an “inverted” TS-3 structure denoted as i-TS-3, and it was subjected TS optimization. In this way, i-TS-3 corresponds to the loss of a H_2_O molecule, while the original TS-3 corresponds to the addition of a H_2_O molecule from the opposite site. The same procedure was applied to IC-2 to generate an “inverted” IC-2 denoted as i-IC-2. A 13.9 kcal/mol energy difference was calculated between i-TS-3 and TS-3 in terms of the ONIOM energy with the 6-31G basis set. This control calculation is in agreement with the previous energy difference between TS-2 and TS-3. This result points out that the different chemical environments play important roles during the transition states.

#### 3.2.3 Intermediate Complex-3 (IC-3)

**Figure 11:**
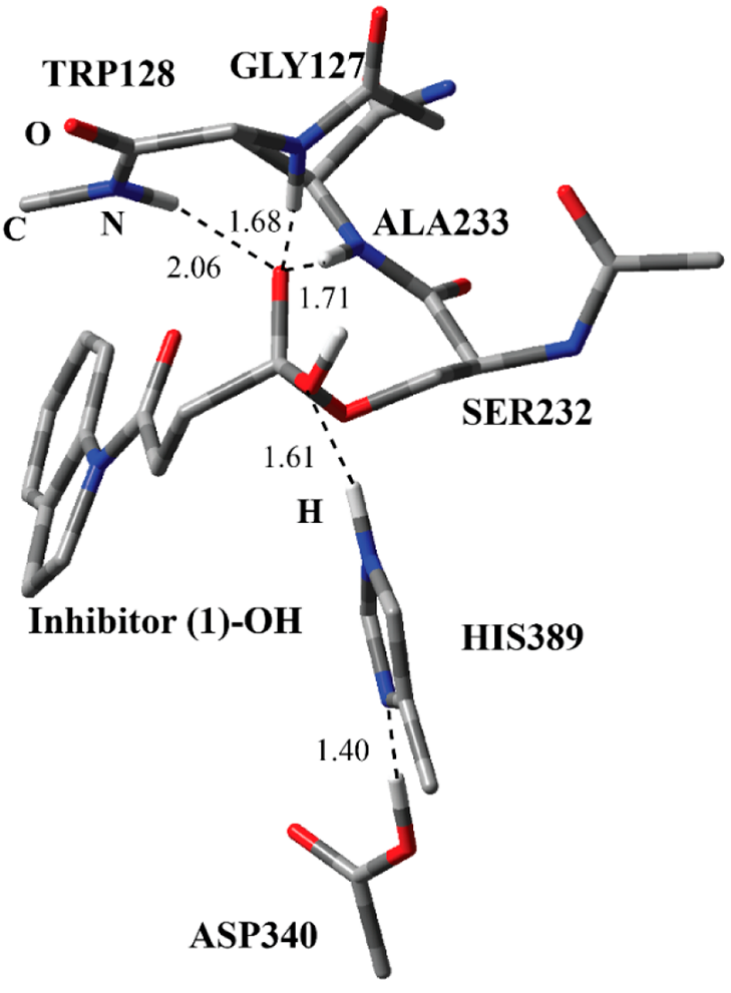
Structure of the optimized IC-3 structure for the first step of the deacylation process including the inhibitor **1**-Ser232-OH tetrahedral intermediate, and catalytically important residues in the QM region obtained with ONIOM (M06-2*X*/6-31G:Amber) with tube models excluding H atoms except the ones shown with an ivory color. The distances are given in angstroms.

The optimized structure of the intermediate complex (IC-3) for the nucleophilic addition of a water molecule to the inhibitor of the carbonyl C atom reveals a tetrahedral oxyanion structure stabilized by three residues through H-bonding interactions (Figure 11). As in the case of the acylation process, the deacylation process was found to proceed through two steps, namely, the nucleophilic addition of a water molecule forming a tetrahedral intermediate and then the elimination of Ser232 forming the carboxylic acid form of the inhibitor and free Ser232 residue. The water molecule was deprotonated by the His389, and Asp340 was protonated by His389. The oxyanion has H-bonding interactions with Trp128, Gly127, and Ala233.

The ONIOM energy difference between TS-3 and IC-3 corresponding to the activation energy of the reverse process was found (7.2 kcal/mol) with the 6-31G basis set (Step 3 in Figure 5). The same barrier was estimated (5.6 kcal/mol) in terms of free energy (Step 3 in Figure S1). Single-point energy calculations with the 6-311+G(2d,2p) basis set estimated a similar activation barrier, 5.9 kcal/mol. 6-311+G(2d,2p) calculations estimated a more endergonic process for Step 3. This is in agreement with calculations in Step 1, which involves the formation of a similar oxyanion intermediate.

#### 3.2.4 Transition State-4 (TS-4)

The transition state of the second step of the deacylation process was obtained using a PES scan similar to the second step of the acylation step. The bond coordinate between the O atom at Ser232 and the carbonyl C atom at the inhibitor was scanned by increasing the distance over a number of steps. The highest energy point was used to locate the transition state corresponding to decomposition of the tetrahedral intermediate complex into a carboxylic acid form of the inhibitor and free Ser232 residue.

The optimized TS structure points out that the tetrahedral intermediate converts into free Ser232 and carboxylic acid upon the protonation of Ser232 by His389. As Ser232 O moves away from the carbonyl C atom of the inhibitor, a proton transfer occurs from His389 to the Ser232 O atom. At the same time, another proton transfers from Asp340 to the N-1 position at His389. The three oxyanion hole residues stabilize the carbonyl O by H-bonding interactions.

The ONIOM energy difference between IC-3 and TS-4 corresponding to the activation energy of the second step of the deacylation process was found (23.0 kcal/mol) in terms of electronic energy (Step 4 in Figure 5) and (17.0 kcal/mol) in terms of free energy (Step 4 in Figure S1). Although Step 2 and Step 4 involve similar processes—the formation of carbonyl products from oxyanion intermediates—the energy level of TS-4 was estimated to be 5.4 kcal/mol more than that of TS2 relative to RC. However, single-point energy calculations with the 6-311+G(2d,2p) basis set estimated an activation barrier of 14.8 kcal/mol. The energy level of TS-4 as well as the activation barrier for Step 4 estimated by 6-311+G(2d,2p) calculations are similar to those of Step 2. These results indicate that the 6-31G basis set presumably overestimates the energy level of TS-4 relative to RC. On the basis of results obtained with both 6-31G and 6-311+G(2d,2p) basis sets, the combined activation barrier for the deacylation process including both Step 3 and Step 4 is greater than the combined activation energy for the acylation process including both Step 1 and Step 2. A higher activation barrier as well as a more stable IC-2 structure than RC might explain the inhibition process.

#### 3.2.5 Product Complex (PC)

**Figure 12:**
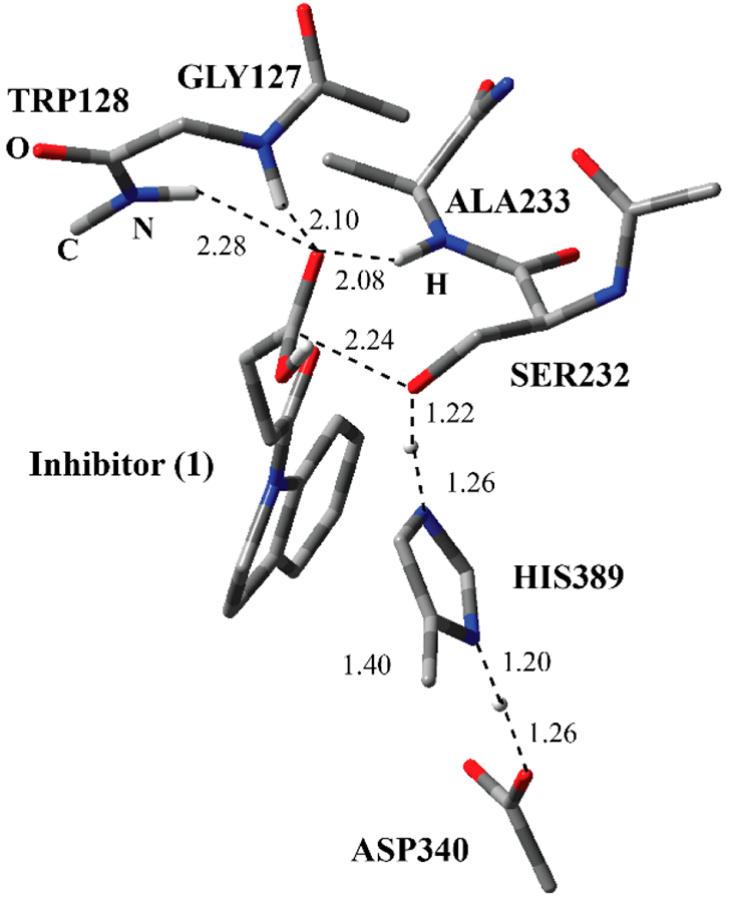
Structure of the optimized TS-4 structure for the second step of the deacylation process including the carboxylic acid form of inhibitor **1** and catalytically important residues in the QM region obtained with ONIOM (M06-2*X*/6-31G:Amber) with tube models excluding H atoms except the ones shown with an ivory color. The distances are given in angstroms.

**Figure 13:**
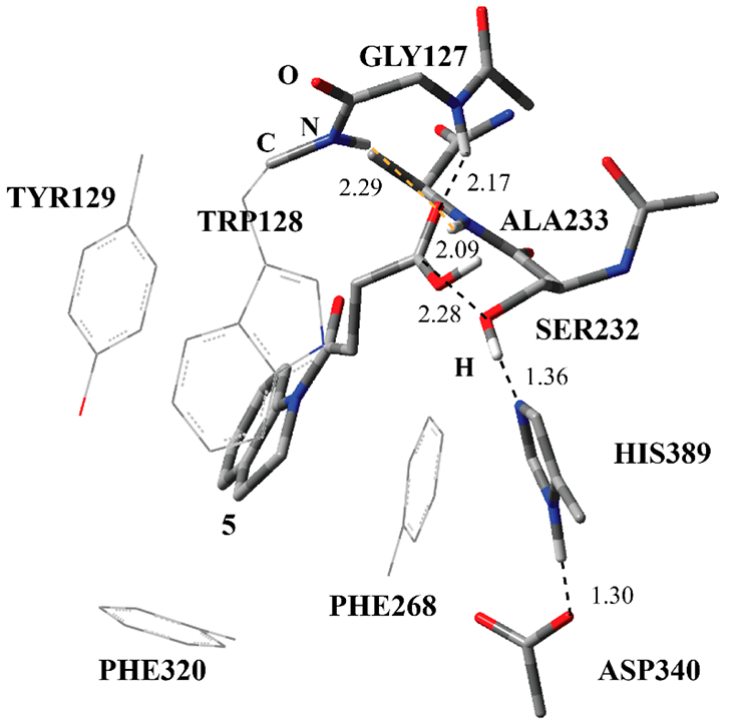
Structure of optimized PC, including the carboxylic acid form of inhibitor **1** (**5**), and catalytically important residues in the QM region belonging to the second step of the deacylation process obtained with ONIOM (M06-2*X*/6-31G:Amber) with tube models excluding H atoms except the ones shown with an ivory color. MM region of Trp128 and three other MM region residues having hydrophobic interactions with **5** are shown in wireframe. The distances are given in angstroms.

The optimization of a proper downhill point in the PES scan of the second step of the deacylation process yielded a product complex (PC) for the overall enzymatic reaction between **1** and Notum (Figure 13). It consisted of a carboxylic acid form of the inhibitor **1** (**5**) and the free Ser232. The ester bond between Ser232 and inhibitor **1** is broken completely. The oxyanion hole residues still maintain their H-bonding interactions with the carbonyl O atom of **5**. However, the H-bonding distances are slightly longer as compared to TS-4 and tetrahedral intermediates, as expected. His389 was protonated by Asp340. All the ionizable residues returned to their native states with respect to RC (Figure 3). The catalytic triad has H-bonding interactions as before in the RC structure. Even though the ester bond between Ser232 and **5** is broken, **5** did not move away considerably from Ser232, and it still maintains all H-bonding and hydrophobic interactions with the surrounding residues. Figure 13 shows that the indole part of **5** is surrounded by the hydrophobic residues, implying that, upon the release of **5**, the PC did not undergo any major rearrangement to release **5**. Furthermore, the distance between the O atom at Ser232 and the carbonyl C atom of **5** did not increase appreciably from TS-4 to PC. In TS-4 the distance between them is 2.24 Å (Figure 12), whereas it is 2.28 Å in PC (Figure 13). This observation suggests that the covalent adduct formed between inhibitor **1** and Ser232 (IC-2 in Figure 9) is stabilized greatly with noncovalent interactions. Especially, the hydrophobic interaction seems to hold the indole part strongly within the active site, and **2** could not move away. From TS-4 to the PC, the H-bonding distances did not increase to a great extent. This also supports the strength of hydrophobic interactions between **5** and surrounding residues such as Tyr129, Phe320, Phe268, and Trp128. The residues shown with wireframe in Figure 13 are in the MM region, and they surround the indole part of **5**.

The energy difference between TS-4 and PC (Figure 6), which corresponds to the ONIOM activation energy of the reverse step for the second of the deacylation process, was calculated (0.7 kcal/mol) in terms of electronic energy and (0.9 kcal/mol) in terms of free energy with the 6-31G basis set. However, single-point energy calculations with the 6-311+G(2d,2p) basis set estimated an activation barrier of 4.9 kcal/mol. Similar to TS-4 calculations, it is highly likely that the 6-31G basis set overestimated the energy level of the PC. However, both basis sets predicted the PC as the least-stable local minimum in the overall catalytic cycle. A high activation barrier for the deacylation process including Step 3 and Step 4 as well as an unstable PC structure point out that the deacylation process is energetically unfavorable and very endergonic. This is presumably as a result of tight binding of the indole part to the active site. This observation is in agreement with the fact that methyl ester **1** acts as an irreversible inhibitor by forming a covalent adduct as a result of transesterification between **1** and Ser232.^14^ On the basis of the crystal structure of this adduct (Figure 2), it was suggested that the deacylation process is unfavorable because of the strong hydrophobic interactions and the unfavorable position of the active site water molecule for the nucleophilic addition step. It was also stated that the nucleophilic attack is hindered, since the angle between the O atom of the water molecule with carbonyl C and O atoms is 87° in the crystal structure (Figure 2); however, the same angle is 114° for the native substrate *O*-palmitoleate ester.^3,14^ On the basis of the optimized geometry of the covalent adduct (Figure 10), we found the same angle (92°), which implies that the in silico adduct is not too different from the crystal structure. The activation barrier for the water addition step (Step 3 in Figure 3 and Figure 5) turned out to be very similar to the first step of acylation process based on 6-31G calculations (Step 1 in Figure 3 and Figure 5). However, single-point energy calculations with the 6-311+G(2d,2p) basis set indicate that the nucleohilic attack of a water molecule requires the highest energy barrier and presumably is the rate-limiting step. This observation is in agreement with the experimental result. However, a high energy level of the PC causing a very endergonic last step should also be one of the main reasons of the inhibition mechanism. It must be stressed that strong hydrophobic interactions of the indole part with the surrounding residues might contribute to the irreversible adduct formation to a significant extent.

## 4 Conclusion

In this study, the inhibition mechanism of Notum by a methyl ester of an indole-based ligand was analyzed with ONIOM calculations using DFT and MM methods using model systems obtained from available crystal structures. The calculations showed that the overall hydrolysis process for the methyl ester occurs in four steps as reported for the other esterase systems.^16^ The catalytic triad causes the deprotonation of Ser232 and water molecule, thereby activating them for the nucleophilic addition steps. The oxyanion intermediates are stabilized by the amide groups of the oxyanion hole residues—Gly127, Trp128, andAla233. It was found that the deacylation process including the last two steps is energetically unfavorable due to strong hydrophobic interactions between the indole part of the inhibitor and surrounding residues. The resulting carboxylic acid and free Ser232 residues kept together forming an unstable product complex. These results provide invaluable insight into the understanding of the inhibition mechanism of Notum as well as the catalytic mechanism of the carboxy esterase family.

## Supporting Information Available

Tables associated with Gibbs free energy, enthalpy, zero-point corrected electronic energies and imaginary frequencies of transition states obtained with ONIOM(M06-2*X*/6-31G:Amber) method; Gaussian input files (gjf file types) of RC, TS-1–4, IC-1–3, and PC species; a figure showing ONIOM electronic and free energies of all species obtained with 6-31G basis set (PDF)
(ZIP)
