# Supplementary material for: Computational Analysis of the Inhibition Mechanism of NOTUM by the ONIOM Method
Source: ACS Omega. 2022 Apr 7;7(15):13333–42. doi: 10.1021/acsomega.2c01044 (PMC9026088; doi:10.1021/acsomega.2c01044)
Supplement: Supplementary file 1 — ao2c01044_si_001.pdf [file ao2c01044_si_001.pdf]

Computational Analysis of Inhibition Mechanism of NOTUM by ONIOM Method

Author(s): Ibrahim Yildiz[†*], Banu Sizirici Yildiz[‡]

[†]Khalifa University, Chemistry Department, PO Box 127788, Abu Dhabi, UAE Tel: +971 (0)2 401 8208

[‡]Khalifa University, CIVE Department, PO Box 127788, Abu Dhabi, UAE

*E-mail: ibrahim.yildiz@ku.ac.ae

Supporting Tables

Table S1. Absolute energies (E), zero-point corrected absolute energies (E+ZPE), enthalpy (H), Gibbs free energies (G) of reactant complex (RC), intermediate complex (IC-1), and transition state (TS-1) together with imaginary frequency of TS-1 for Step 1

**Species**	**E (au)**	**E+ZPE (au)**	**H (au)**	**G (au)**	**Imaginary Frequency (*i*)**
**RC**	-2592.03	-2583.82	-2583.29	-2584.43	
**TS-1**	-2591.99	-2583.79	-2583.27	-2584.40	-229.04
**IC-1**	-2592.00	-2583.79	-2583.26	-2584.40	

Table S2. Absolute energies (E), zero-point corrected absolute energies (E+ZPE), enthalpy (H), Gibbs free energies (G) of intermediate complex (IC-1), transition state (TS-2), and intermediate complex (IC-2) together with imaginary frequency of TS-2 for Step 2

**Species**	**E (au)**	**E+ZPE (au)**	**H (au)**	**G (au)**	**Imaginary Frequency (*i*)**
**IC-1**	-2592.00	-2583.79	-2583.26	-2584.40	
**TS-2**	-2591.97	-2583.77	-2583.25	-2584.38	-273.37
**IC-2**	-2592.02	-2583.81	-2583.29	-2584.42	

Table S3. Absolute energies (E), zero-point corrected absolute energies (E+ZPE), enthalpy (H), Gibbs free energies (G) of intermediate complex (IC-2), transition state (TS-3), and intermediate complex (IC-3) together with imaginary frequency of TS-3 for Step 3

**Species**	**E (au)**	**E+ZPE (au)**	**H (au)**	**G (au)**	**Imaginary Frequency (*i*)**
**r-IC-2**	-2478.07	-2466.32	-2465.56	-2467.16	
**TS-3**	-2478.05	-2466.30	-2465.54	-2467.15	-375.85
**IC-3**	-2478.07	-2466.31	-2465.55	-2467.16	

Table S4. Absolute energies (E), zero-point corrected absolute energies (E+ZPE), enthalpy (H), Gibbs free energies (G) of intermediate complex (IC-3), transition state (TS-4), and product complex (IC-3) together with imaginary frequency of TS-3 for Step 4

**Species**	**E (au)**	**E+ZPE (au)**	**H (au)**	**G (au)**	**Imaginary Frequency (*i*)**
**RC1**	-2478.06	-2466.31	-2465.55	-2467.16	
**TS2**	-2478.02	-2466.28	-2465.52	-2467.13	-246.33
**PC2**	-2478.03	-2466.28	-2465.52	-2467.13	

Supporting Figures

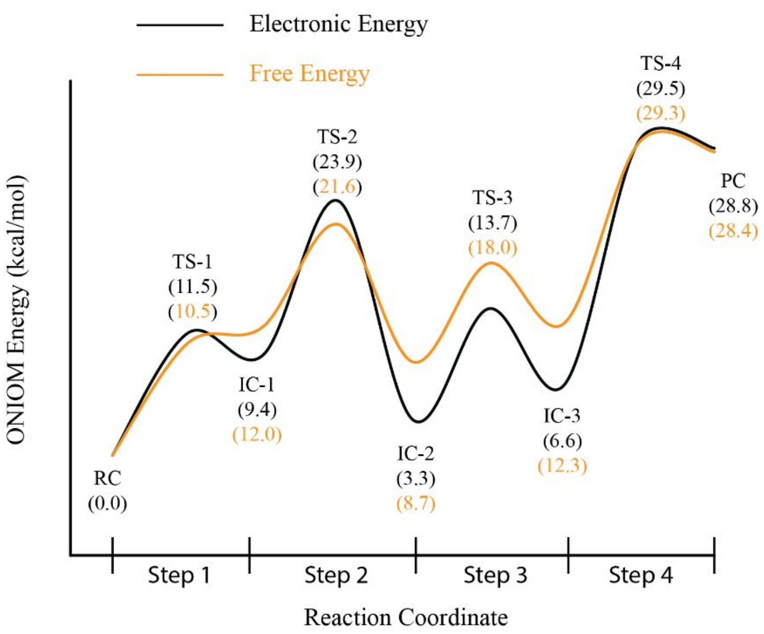

Figure S1. The energy profile for the acylation (Step 1 and 2) and deacylation processes (Step 3 and 4) of the inhibitor **1** by Notum obtained with ONIOM(M06-2X/6-31G:Amber) method. (The values in the parentheses with black color refers to the ONIOM electronic energies of each complexes in kcal/mol relative to the initial reactant complex (RC), and the values in the parentheses with orange color refers to the ONIOM free energies of each complexes in kcal/mol relative to the initial reactant complex (RC)).
